# Frequency of maintenance hemodialysis patients meeting K/DOQI criteria for serum calcium, phosphorus, calcium phosphorus product and PTH levels; a single institutional experience from Pakistan: a cross sectional study

**DOI:** 10.11604/pamj.2019.33.183.18057

**Published:** 2019-07-09

**Authors:** Taimoor Khalid Janjua, Kunwer Naveed Mukhtar, Ahmed Kunwer Naveed, Erum Bashir Ahmed, Muhammad Rehan

**Affiliations:** 1 Liaquat National Hospital and Medical College, National Stadium Road, Karachi, Pakistan; 2 Dow University of Health Sciences, Karachi, Pakistan

**Keywords:** Maintenance hemodialysis, K/DOQI criteria, serum calcium, frequency, ESRD patients

## Abstract

**Introduction:**

There is a great scarcity of literature in Pakistan investigating the proportion of end stage renal disease (ESRD) patients undergoing hemodialysis (HD) who meet the recommended kidney diseases outcome quality initiative (K/DOQI) guidelines for serum calcium (Ca), phosphorus (P), calcium phosphorus product (Ca x P) and parathyroid hormone (PTH) levels. Our study aimed to determine frequencies of patients who met the K/DOQI targets for these minerals at a tertiary care hospital's dialysis unit.

**Methods:**

111 ESRD patients on maintenance HD were selected from a tertiary care hospital. Serum Ca and P were assayed on chemistry analyser. PTH was measured through electrochemiluminescence sandwich method. Data were compared with K/DOQI targets and analysed using SPSS-21.

**Results:**

The mean age of patients was 55.85 years (SD ± 13.95). Gender distribution was almost equal with 49.5% males and 50.5% females. The patients had mean corrected serum Calcium 9.12 ± 0.64 mg/dL, Phosphorus 4.57 ± 1.54 mg/dL and Parathyroid hormone 333.8 ± 278.4 pg/mL. The patients had achieved K/DOQI target ranges of Ca, P, PTH, Ca x P product and all 4 criteria in 63.1%, 47.6%, 38.7%, 84.7% and 10.8% respectively.

**Conclusion:**

Majority of patients on maintenance HD at our institution did not achieve the recommended K/DOQI target ranges. Further studies pertaining to the Asian subcontinent will prove resourceful for comparison of mineral metabolism and dialysis outcome of ESRD patients.

## Introduction

Epidemiological data shows that about 10% of the worldwide population is affected by chronic kidney disease (CKD) and millions die each year since they cannot access affordable treatment [1]. Over two million people worldwide currently receive treatment through dialysis or a kidney transplant to stay alive, yet this number may only represent 10% of the people who actually need treatment to live [2,3]. In Pakistan the burden of CKD is enormous as well. Approximately 25% of 300 subjects who came to a screening camp were found to have CKD [4]. Complications of CKD cover a diverse plethora of systemic disorders, notably increased cardiovascular mortality, cognitive decline, anaemia, mineral and bone disorders, and fractures [5]. Maintenance dialysis is thus of utmost importance for end stage renal disease (ESRD) patients [6]. Their serum Calcium (Ca), Phosphorus (P) levels and Calcium-Phosphorus product (Ca x P) are essential indicators for monitoring bone mineral disease. Therefore, patients of CKD and ESRD undergoing Maintenance HD (MHD) require constant monitoring. The Kidney Diseases Outcome Quality Initiative (KDOQI) clinical guidelines for hemodialysis (HD) are a useful criteria which help to monitor serum Ca, P and parathyroid hormone (PTH) levels in CKD patients [7]. According to a multi-centre study conducted in Korea it was concluded that despite the current clinical practice guidelines, Secondary Hyperparathyroidism (SHPT) seems to be inadequately controlled in many Maintenance HD (MHD) patients. Uncontrolled SHPT was associated with higher levels of serum Ca, P and Ca × P product, suggestive of the importance of SHPT management [8]. Asiatic [8,9], local [10] and various international studies [11-16] have been conducted in different countries to assess mineral metabolism abnormalities in ESRD patients receiving hemodialysis in concordance with the K/DOQI guidelines [7]. Multi centre dialysis outcome and practice pattern studies (DOPPS I and DOPPS II) were conducted at two point intervals in patients on hemodialysis in France, Germany, Italy, Japan, Spain, United States and the United Kingdom. The DOPPS I study provided the baseline information regarding the prevalence of Ca, P and PTH metabolism abnormalities among hemodialyzed patients with reference to K/DOQI targets [14]. DOPPS II revealed marked improvement in the bone mineral parameters as compared with the previous DOPPS I study over time [14]. These two large studies serve as the initial “yard stick” for local dialysis centres initially wishing to compare their adequacy. However, there is still a great paucity regarding comparison with local and Asiatic literature. The purpose of this study was to determine the frequency of ESRD patients undergoing Maintenance HD at our institution who met the K/DOQI Criteria for serum Ca, serum P, Ca x P product and PTH levels.

## Methods

This cross-sectional study was conducted at the Dialysis Unit of the Nephrology Department at Liaquat National Hospital and Medical College. Respective approvals were obtained from the Research and Ethics Committees of the Institute. The study was conducted from July 2017 till January 2018. After obtaining informed consent we enrolled one hundred and eleven patients with creatinine clearance value < 15 ml/min/1.73m^2^ of either sex above 12 years of age. Demographic data, history of illness and detailed physical examination were recorded. Consent, privacy and confidentiality was maintained for all patients as per the Helsinki Declaration. Patients were receiving maintenance hemodialysis at once, twice or in thrice weekly sessions for at least the last three months and taking Vitamin D, Calcium Supplements to control calcium levels, either CaCO3 or CaAc as phosphate binders to control hyperphosphatemia and One-alpha-hydroxy-cholecalciferol for PTH regulation. Patients suffering from granulomatous diseases, acute renal failure, on peritoneal dialysis, and having pervious Para thyroidectomy were excluded from the study. Five ml pre-dialysis blood was drawn in Li-heparin tubes and plasma separated by centrifugation at 5000rpm. Plasma levels of Calcium, inorganic phosphate and albumin were assayed by using 3^rd^ generation kits from Roche Diagnostics (Roche Diagnostics, Basil) on chemistry auto-analyzer Cobas c501 (Roche Diagnostics, Basil). Calcium was estimated by NM-BAPTA method [17] whereas inorganic phosphate and Albumin were estimated by Phospho-Molybdate [18] and Bromocresol green methods [19], respectively. Additionally, intact PTH was determined by second-generation iPTH assay by electrochemiluminescence (ECL) sandwich method on Immunoassay analyzer Cobas e411 (Cobas, Roche Diagnostic, Basil) [20]. CV for all biochemical parameters and PTH were within 2-5%. All patients clinically labelled as having ESRD met the inclusion criteria for the study. Patients having active tuberculosis or related granulomatous diseases, carcinoma, endocrine disorders involving the thyroid and parathyroid gland were excluded from the study. Since it was not a primary objective of this study to identify or investigate medication compliance/adherence we reported them as observational findings. Medication compliance/adherence for vitamin D, calcium supplements was defined as being present in those ESRD patients who presently admitted fully resorting to their prescribed dosages since initiation of hemodialysis. K/DOQI 2005: serum calcium between 8.4 and 9.5mg/dL, phosphorus concentration between 3.5 and 5.5mg/dL, phosphocalcium product <55mg^2^/dL^2^, intact parathyroid hormone between 150 and 300pg/ml [7].

**Data analysis procedure**: statistical package for social sciences SPSS 21(Chicago, IL, USA) was used for data compilation and analysis. Frequencies, percentages, mean and standard deviation (SD) were calculated for quantitative variables (age, serum mineral levels and related parameters). Frequencies and percentages were calculated for qualitative variables (percentage (%) of patients who met KDOQI and KDIGO guideline targets).

## Results

Among 111 ESRD patients the mean age of patients was 55.85 years (SD ± 13.95 years). Gender distribution was almost equal with 55 (49.5%) males and 56 (50.5%) females. The mean dialysis duration was 36.28 months (SD±57.16 months) with majority patients having long term follow up. The mean distribution of minerals has been illustrated in Table 1, as per K/DOQI guidelines in Table 2 and KDIGO guidelines in Table 3. Upon analyzing frequencies, we found that majority hyper-phosphatemic patients (55%) had higher PTH levels. After excluding data for 21 (19%) patients due to non-compliancy it was found that three weekly cycle patients were most common (59.9%) followed by twice weekly (35.2%) and once a week (4%). All remaining patients (81%) strictly adhered to their dialysis schedule. Observational findings: compliancy regarding vitamin D and Calcium supplement intake was only observed in about half (50%) of the patients who were prescribed these supplements.

**Table 1:** Mean distribution of minerals

Parameter	Mean(±SD)	Median	Range
Total Calcium(mg/dL)	9.12(±0.64)	9.14	6.98-11.40
Phosphate(mg/dL)	4.57(±1.50)	4.70	1.20-9.30
Calcium X Phosphorus(mg^2^/dl^2^)	41.53(±12.74)	42.25	10.33-80.69
PTH(pg/mL)	334.8(±278.4)	256	11.0-2104

**Table 2:** Distribution of patients as per K/DOQI targets

Parameter	Number of Patients (Percentage %) (All n=111)
**Total Calcium(mg/dL)**	
<8.4	15(13.51)
8.4-9.5*	70(63.06)
>9.5	26(23.42)
**Serum Phosphorus(mg/dL)**	
<3.5	29(26.16)
3.5-5.5*	53(47.74)
>5.5	29(26.16)
**Calcium Phosphorus Product(mg^2^/dL^2^)**	
<55*	94(84.68)
>55	17(15.31)
PTH(pg/mL)	
<150	23(20.70)
150-300*	43(38.73)
>300	45(40.54)
All 4 Criteria	12(10.8)

* K/DOQI targets

**Table 3:** Distribution through KDIGO guidelines

Parameter	Number of Patients (Percentage %) All (n = 111)
**Total Calcium(mg/dL)**	
<8.4	13(11.71)
8.4-10.4*	96(86.48)
>10.4	2(1.80)
**Serum Phosphorus(mg/dL)**	
<3.2	18(16.21)
3.2-6.0*	78(70.21)
>6.0	15(13.51)
**PTH (pg/mL)**	
<134	16(14.41)
150-603*	83(74.77)
>603	12(10.81)

* KDIGO targets

## Discussion

Among our patients, 63.1% met the KDOQI criteria for albumin corrected serum Ca levels. Lesser patients (47.6%) met the serum P levels target. Even lesser (38.7%) met the PTH level targets. A majority (84.7%) met the targets for Ca x P. 10.8% patients met all four targets altogether while only four (3.6%) patients failed to meet any KDOQI target. Using albumin corrected Ca levels more patients displayed higher versus lower Ca levels (23.4% v 13.5%). Regarding P levels, patients equally displayed higher versus lower levels (26.2% v 26.2%). More patients displayed higher versus lower PTH levels (40.6% v 20.7%). Our findings showed general improvements for serum Ca, P, Ca x P and PTH targets respectively compared to the findings from the DOPPS I [13] (40.5%,40.8%,56.6%,21.4%), DOPPS II [13] (42.5%,44.4%,61.4%,26.2%) and a large Asian [Japanese (J-DOPPS)] study [8] (44.2%,43.1%,58.9%,24.4%). As is evident from our comparisons through Table 4, patients from our study meeting KDOQI targets were greater in proportion than several other Asiatic [9], local [10] and international [11-16] studies. Upon local comparison [10] our findings were improved for all parameters. Our Ca x P product showed improved levels in comparison to all above studies. Only Maudell F *et al*. [13] reported improved findings for serum Ca targets while for serum P our study only showed improved findings in contrast to the findings of Dilshad *et al*. [10], Young EW *et al*. [14] and Benabdellah *et al*. [16]. Only Rivera F *et al*. [12] reported better outcomes for all four KDOQI targets.

**Table 4:** Comparison with other studies involving K/DOQI targets

Parameters									
Total Calcium (mg/dL)		Kim GH *et al.* 2014 [9]	Dilshad *et al*. 2009 [10]	Lebner *et al*. 2010 [11]	Rivera F *et al*. 2006 [12]	Maudell F *et al*. 2005 [13]	Young EW *et al*. 2004 [14]	Djukanović *et al*. 2016 [15]	Benabdellah *et al*. 2013 [16]
<8.4	13.5		16.8	34.8	9.1	5	9	_	_
8.4-9.5	63.1	58.7	37.9	58.6	53.7	77.2	41	53.2	42.2
>9.5	23.4		45.3	6.6	37.1	17.8	50	_	_
**Serum Phosphorus (mg/dL)**									
<3.5	26.2		5.3	15.9	11.7	16	8	_	_
3.5-5.5	57.6	50.7	41	59.7	57.2	55	40	49.5	46.9
>5.5	26.2		53.7	24.4	31.1	29	52	_	_
**Calcium Phosphorus Product (mg^2^/dL^2^)**									
<55	84.7	70.7	61.5	83.3	76.4	73	56	_	78.1
>55	15.3	29.3	38.5	16.7	23.4	27	44	_	21.9
**PTH (pg/L)**									
<150	20.7	42.7	30	27.2	25.1	43	51	_	29.7
150-300	38.7	30.8	22.6	29.7	31.1	26	22	21	20.3
>300	40.6	26.5	27.4	43.1	43.7	31	27	_	51
**All 4 Criteria**	10.8		6.9	11.7	25	7.3	_	_	8.4

*K/DOQI targets

Comparison of patients meeting K/DOQI targets (stated in bold) with local [9] and international studies [10-16]

Differences with international populations can mostly be owed to genetic and ethnic variations. Improvements over local and Asiatic populations can be owed firstly to more diligent care on part of the health clinicians, which is in turn attributed to the increasing global burdens of CKD [1]. Secondly improved medications particularly of PTH, vitamin D and calcium phosphate binders have greatly improved outcomes for ESRD patients. Differing dietary intakes and habits can also partly explain result variations. Overtime chronic kidney disease contributes to development of osteodystrophy [21] and thus increased PTH levels. Similar to a local study [10] majority of our patients with higher P levels (55.2%) also had increased PTH levels. Chronic HD patients thus require closer monitoring for PTH levels. Our institute receives patients from across the whole country. We can expect results from dialysis setups nationwide to be better or worse. Local clinicians should ensure policies for regular testing at their respective nationwide dialysis units to monitor and decrease the frequency of deranged mineral levels, particularly those lacking comprehensive National Health Insurance policies. For comparison purposes, results have also been included according to the 2009 KDIGO guidelines [22] in Table 3. The differing results for the KDOQI and KDIGO guidelines need to be addressed and investigated further.

**Limitations of the study**: our single institutional study is limited by a relatively smaller sample size, the specific inclusion of maintenance HD patients and the exclusion of medication usage and effects as an independent investigative variable owing to the non-adherent and inequitable attitude of majority patients. We used total corrected Calcium levels for comparison. Some studies did not specify which Calcium levels were used. To allow effective and fair comparison, the 2005 K/DOQI guidelines have been used. Impressions of carelessness were evident from our patients regarding medication intakes excluding those pertaining to injectable and expensive medications. Only about half of the patients who were prescribed Vitamin D and Calcium supplements admitted full compliancy. It was thus very difficult in our study to quantify and investigate medication usage/compliance as an independent variable with respect to mineral targets and metabolism. Hence, they should be viewed as observational findings only.

## Conclusion

Determining mineral derangements in ESRD HD patients is of paramount importance for developing nations. On the basis of single institutional comparisons locally, a moderate improvement over time in mineral metabolism was seen with regards to the K/DOQI criteria. Although this is appreciable, the greater room for improvement must be addressed. We recommend further that, such institutional studies be conducted internationally and particularly among local ESRD patients, so as to reveal a bigger picture and also to understand underlying causes for similarities and differences between the K/DOQI and KDIGO guidelines.

### What is known about this topic

- Previous studies have reported varying proportions of patients undergoing HD meeting the K/DOQI guidelines for key mineral metabolism;
- The proportion of patients meeting the criteria and targets has generally been low over the last couple of decades.

### What this study adds

- Our local study adds a recent data from a Pakistan population to the currently available literature;
- Our findings illustrate that still few patients meet the K/DOQI criteria for ESRD patients;
- Our findings illustrate differences between patients meeting KDIGO and KDOQI guidelines and the need to address them.

## Competing interests

The authors declare no competing interests.
